# Non-Orthogonal Multiple Access with One-Bit Analog-to-Digital Converters Using Threshold Adaptation

**DOI:** 10.3390/s23136004

**Published:** 2023-06-28

**Authors:** Moonsik Min, Jae-Ik Kong, Tae-Kyoung Kim

**Affiliations:** 1 School of Electronics Engineering, Kyungpook National University, Daegu 41566, Republic of Korea; msmin@knu.ac.kr; 2 School of Electronic and Electrical Engineering, Kyungpook National University, Daegu 41566, Republic of Korea; te04034@knu.ac.kr; 3 Department of Electronic Engineering, Gachon University, Seongnam 13120, Republic of Korea

**Keywords:** one-bit analog-to-digital converter, interference cancellation, multiple access channel, successive detection, power allocation

## Abstract

In digital communication systems featuring high-resolution analog-to-digital converters (ADCs), the utilization of successive interference cancellation and detection can enhance the capacity of a Gaussian multiple access channel (MAC) by combining signals from multiple transmitters in a non-orthogonal manner. Conversely, in systems employing one-bit ADCs, it is exceedingly difficult to eliminate non-orthogonal interference using digital signal processing due to the considerable distortion present in the received signal when employing such ADCs. As a result, the Gaussian MAC does not yield significant capacity gains in such cases. To address this issue, we demonstrate that, under a given deterministic interference, the capacity of a one-bit-quantized channel becomes equivalent to the capacity without interference when an appropriate threshold value is chosen. This finding suggests the potential for indirect interference cancellation in the analog domain, facilitating the proposition of an efficient successive interference cancellation and detection scheme. We analyze the achievable rate of the proposed scheme by deriving the mutual information between the transmitted and received signals at each detection stage. The obtained results indicate that the sum rate of the proposed scheme generally outperforms conventional methods, with the achievable upper bound being twice as high as that of the conventional methods. Additionally, we have developed an optimal transmit power allocation algorithm to maximize the sum rate in fading channels.

## 1. Introduction

In modern wireless communication systems, digital signal processing (DSP) is essential to ensure high speed and reliable communication [1,2]. In practice, an inevitable quantization error occurs in analog-to-digital converters (ADCs), which may cause a mismatch between theory and implementation, because most digital communication theories were derived assuming the use of infinite-resolution ADCs. As a result, traditional communication standards, such as long-term evolution, recommended using high-resolution ADCs in receivers.

To satisfy the consistently increasing demand for data rates, recent standards for wireless communication are focusing on utilizing large amounts of unused bandwidth in the millimeter-wave (mmWave) and terahertz frequency bands [3,4,5,6,7,8,9,10,11,12,13]. In general, they try to accommodate the fast-growing demand for higher data rates by leveraging the significantly wider bandwidth available in higher-frequency bands, such as the mmWave band. However, the use of such wider bandwidths can lead to the dissipation of a substantial amount of power when employing conventional high-resolution ADCs. This is because the power consumption of ADCs increases linearly with higher sampling rates [14]. Power consumption is a critical issue for mobile devices, as it directly affects the battery lifetime and available power resources for uplink communications.

Receiver structures based on low-resolution ADCs have garnered significant attention as a useful solution for exploiting the abundant resources available in extremely high-frequency bands while achieving low power consumption [15]. In particular, the utilization of one-bit ADCs as the receivers has been widely studied as an extreme case due to its high power efficiency. Extensive research has been dedicated to evaluating the performance of communication channels with one-bit ADCs [15,16,17,18,19,20,21,22,23,24,25,26,27,28,29,30].

The Shannon theoretic capacity of a single-input and single-output (SISO) real-valued additive white Gaussian noise (AWGN) channel with one-bit ADCs was derived in [16]. Building upon this, the capacity of complex fading channels with multiple transmit and receive antennas was considered in [17]. For instance, assuming perfect channel state information at the transmitter, the capacity of a multiple-input and single-output (MISO) complex fading channel was derived for the given channel components. Furthermore, various upper and lower bounds have been presented for multiple-input and multiple-output (MIMO) complex channels. Additionally, numerous important studies have derived the capacities of different wireless channels with one-bit ADCs [18,19,20,21]. In [18], the trade-off between achievable rates and energy rates was considered when employing one-bit ADC receivers. The performance of a one-bit quantized channel with limited channel state information at the transmitter (CSIT) was investigated in [19]. Moreover, the use of one-bit digital-to-analog converters (DACs) in transmitters was explored in [20]. The study in [20] also proposed a channel training method to improve the poor performance of direct channel feedback with one-bit ADCs. The secrecy capacity of a Gaussian wiretap channel with one-bit ADCs was analyzed in [21]. Furthermore, the detection performance of fading channels with one-bit ADCs was studied in [22,23,24]. There have also been several studies in the case of massive MIMO [25]. In [25], a one-bit ADC was used for an uplink massive MIMO system, and the corresponding throughput was analyzed. The paper [26] investigated a decode-and-forward relay protocol using one-bit ADCs. In [27], authors analyzed the performance of angle of arrival (AoA) estimation while considering the use of one-bit ADCs and DACs at the transmitter and receiver, respectively. In addition, the performance of AoA estimation using a massive uniform linear array was analyzed in [28].

In addition to the studies on the performance of point-to-point channels with one-bit ADCs, several studies have recently focused on the capacity of multiple access channels (MACs) with one-bit ADCs. For example, researchers [31,32,33] have studied one-bit ADC-based Gaussian MAC, particularly those in which two transmitters communicate with a single receiver. In [31], a MAC with AWGN was considered and the capacity region was derived. The authors also proved that the boundary points of the capacity region can be achieved by discrete input distributions, so it suffices to consider only discrete random variables as inputs to the MAC with AWGN. In [32], a two-transmitter MAC was considered in Rayleigh fading environments with AWGN. Here, the authors proposed a signaling scheme and the corresponding input distribution (circularly symmetric with bounded amplitude) and proved that their scheme can achieve the capacity region under the premise of perfect channel state information at the receiver (CSIR). In [33], the capacity region of MAC in the presence of Gaussian-mixture co-channel interference was analyzed. These studies contribute to the understanding of the capacity limits and achievable performance of MACs with one-bit ADCs, considering different channel conditions and interference scenarios.

If the receiver uses infinite-resolution ADCs (IR-ADCs), DSP techniques such as successive interference cancellation (SIC) can effectively eliminate non-orthogonal interference. As a result, the channel capacity of a two-transmitter MAC can be twice as high as that of a SISO channel when the transmit power is sufficient [2]. However, with one-bit ADCs, the capacity has the same upper bound as the SISO capacity [31,32,33], although we have a sufficient transmit power. This is because non-orthogonal inter-signal interference (ISI) between different transmitters cannot be adequately removed using DSP techniques such as SIC due to the nonlinear distortion introduced by one-bit ADCs. Consequently, the performance of DSP-based techniques, such as SIC, is compromised in the presence of one-bit ADCs.

To address the capacity limitation in a MAC with one-bit ADCs, an alternative interference cancellation technique based on threshold adaptation is proposed in this study. This novel technique aims to eliminate non-orthogonal ISI between different transmitters in a two-transmitter MAC with one-bit ADCs. By effectively mitigating the non-orthogonal ISI, the proposed scheme has the potential to achieve a capacity that is twice as high as conventional approaches, particularly in a high signal-to-noise ratio (SNR) regime. By incorporating successive detection after the proposed interference cancellation process, the capacity scaling is achieved, leading to significantly improved performance. This study also provides analytical derivations for the achievable rate region of the proposed scheme, offering a theoretical understanding of its performance. Simulation results are also presented, confirming that the proposed scheme achieves significantly higher sum rates compared to conventional schemes. This performance improvement is attributed to the benefits gained from the proposed interference cancellation technique. The main contributions of this study can be summarized as follows:

- A SIC scheme operating in the analog domain is proposed for a two-transmitter MAC with AWGN. Achieving substantial gain using SIC in the digital domain is nearly impossible when using one-bit ADCs due to nonlinear distortion caused by one-bit quantization. The proposed scheme addresses this issue by incorporating analog domain threshold adaptation. The achievable rate of the proposed scheme is theoretically analyzed, demonstrating its significant performance superiority over conventional methods.
- The maximum achievable rate of the proposed scheme can be up to twice as high as the capacity of a SISO channel in the high SNR regime, even when the total transmit powers are equal for both systems. This observation differs from the scenario where SIC is performed with IR-ADCs, where the capacity of a two-transmitter MAC is equivalent to that of a SISO channel if the total transmit powers for both systems are equal. This implies that the impact of using SIC is much more significant in systems with one-bit ADCs compared to systems with IR-ADCs.
- When the total transmit power is constrained, the achievable rate of the proposed scheme depends on the ratio between the received signal powers from different transmitters. Furthermore, in wireless fading channels, the realizations of fading channel gains significantly impact the received signal power ratio. Consequently, the instantaneous sum rate is greatly influenced by the power allocation between the two transmitters. Therefore, this study also introduces a power allocation algorithm tailored to the specific channel conditions. The algorithm effectively maximizes the achievable rate of the proposed system in wireless fading channels.

The remainder of this paper is organized as follows. Section 2 describes the system model and preliminaries, Section 3 presents the proposed SIC with successive detection, and Section 4 considers an extension to wireless fading channels. Section 5 introduces a lookup table-based optimal power allocation strategy that is well-suited for the proposed method. It also presents simulation results to validate the effectiveness of the proposed approach. Finally, Section 6 concludes the paper and summarizes the key findings.

## 2. System Model and Preliminaries

### 2.1. System Model

In this study, we consider the two-transmitter and single-receiver memoryless AWGN MAC. The received signal of the channel is represented as [31,32]

(1) $$\begin{array}{c} Y=X_{1}+X_{2}+W, \end{array}$$

where *Y* is the analog domain received signal, $X_{1},X_{2}$ are the transmit signals from two different transmitters, and *W* represents AWGN with zero mean and unit variance (noise variance is normalized to 1 for simplicity). The received signal *Y* is connected to a one-bit resolution quantizer, which is modeled using the function $f_{Q}\left(x,\tau \right)$, that maps the real-valued input *x* to one of two output symbols using threshold τ as follows:

(2) $$\begin{array}{c} f_{Q}\left(x,\tau \right)=\left\{\begin{array}{cc} 1, & x\geq \tau \\ -1, & x<\tau \end{array}\right.. \end{array}$$

We initially analyze a two-transmitter MAC with AWGN in (1). Additionally, in Section 4, we extend our analysis to complex wireless fading channels for a more realistic and practical discussion.

### 2.2. Related Work

In this subsection, we provide a summary of previous studies conducted on the two-transmitter MAC with AWGN. These studies will serve as a comparison group to showcase the advantages of the method proposed in this study. In particular, the work by Mo et al. [17] derived the capacity of SISO and MISO complex fading channels with one-bit ADCs when CSIT is available. Each capacity was derived using the following equations.

(3) $$\begin{array}{c} C_{\mathrm{SISO}}=2(1-H_{b}\left(Q(|H\right|\sqrt{P_{T}}))), \end{array}$$

where $H_{b}\left(\cdot \right)$, Q(·), and *H* denote binary entropy, Gaussian *Q*-function, and channel gain, respectively, and $P_{T}$ denotes the total transmit power. In [31], the capacity region of a normal memoryless AWGN MAC with one-bit ADCs was investigated. The authors utilized an auxiliary random variable and demonstrated that finite and discrete input distributions can achieve the boundary points of the capacity region. Furthermore, in [32], it was shown that an input distribution with bounded amplitudes, specifically a $\frac{\pi }{2}$ circularly symmetric distribution, is an optimal input distribution for the considered system. It is worth noting that this optimal distribution is also finite and discrete, as mentioned previously. The corresponding sum-capacity of a two-transmitter MAC, assuming perfect CSI at the receiver, is derived as (4):

(4) $$\begin{array}{c} C_{\mathrm{MAC},\;\mathrm{CSIR}}=2-E\left[H_{b}\left(|\frac{\sum_{i=1}^{2}\left|H_{i}\right|\sqrt{P_{i}}\cos\left(\phi_{H_{i}}\right)}{\sqrt{0.5}\sigma }|\right)+H_{b}\left(|\frac{\sum_{i=1}^{2}\left|H_{i}\right|\sqrt{P_{i}}\sin\left(\phi_{H_{i}}\right)}{\sqrt{0.5}\sigma }|\right)\right], \end{array}$$

where $H_{i}=\left|H_{i}\right|e^{\phi_{H_{i}}}$ is complex fading channel gains between the receiver and transmitters *i*, and $P_{i}$ is the average transmit power $E[|X_{i}{|}^{2}]$ of transmitter *i*, for i=1,2. Please note that, based on the input distributions examined in previous studies [31,32,33] concerning the two-transmitter MAC, the sum rate is upper-bounded by 1 in real channels and by 2 in complex channels. The upper bound is equal to that of the SISO channels.

## 3. Proposed Scheme: Method and Analysis

### 3.1. Proposed Interference Cancellation and Detection

**Figure 1:**
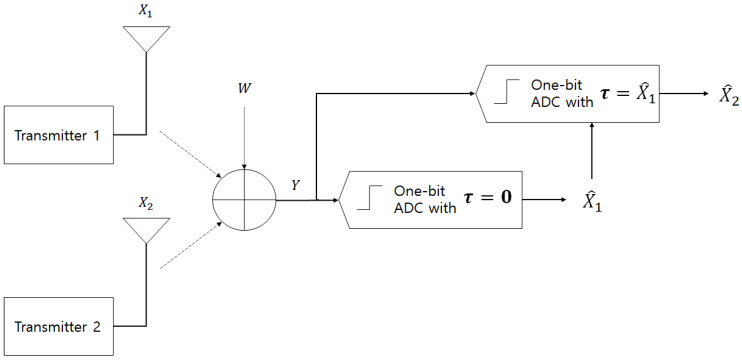
Proposed scheme.

In this study, we propose a successive interference cancellation and detection structure based on a novel interference cancellation scheme designed by adapting the threshold of one-bit ADC. Based on the previous results in [16,17], $X_{1}$ and $X_{2}$ are obtained following binary phase shift keying (BPSK) signaling. At the first stage of the proposed scheme, the receiver detects one transmitter (it can be either $X_{1}$ or $X_{2}$) signal regarding the other transmitter signal as an interference; the threshold value of the corresponding ADC is zero (Figure 1). At the second stage, the previously detected signal is used as a threshold value for the additional one-bit ADC.

Without loss of generality, $X_{1}$ can be detected first, followed by $X_{2}$ at the second stage, as depicted in Figure 1. The achievable rate of transmitter 1 and 2 can be obtained as follows.

**Lemma** **1.** *Suppose that $X_{1}$ and $X_{2}$ are obtained by BPSK signaling and the one-bit ADC at the first stage uses a zero threshold. The error-free achievable rate of transmitter 1, which is denoted as $R_{1}$, is given by*

(5) $$\begin{array}{cc} R_{1}\; & =1-H_{b}\left(\frac{Q(\sqrt{P_{1}}-\sqrt{P_{2}})}{2}+\frac{Q(\sqrt{P_{1}}+\sqrt{P_{2}})}{2}\right), \end{array}$$

*where $H_{b}\left(\cdot \right)$ and Q(·) denote binary entropy and Gaussian Q-function, respectively, and $P_{i}$ is the average transmit power of $X_{i}$ for i=1,2. Moreover, in the derivation, $P_{1}$ and $P_{2}$ in (5) correspond to the received SNRs of the signals from transmitters 1 and 2, respectively.*

**Proof.**  See Appendix A. □

**Lemma** **2.** *For a given value of $X_{1}$, the error-free achievable rate of transmitter 2 is $R_{2}=1-H_{b}\left(Q\left(\sqrt{P_{2}}\right)\right)$, when the threshold value is set as $\tau =X_{1}$ at the second stage.*

**Proof.**  Based on the hypothesis of the lemma, let $Z_{2}=f_{Q}\left(X_{1}+X_{2}+W,X_{1}\right)$. Since $X_{2}$ is detected at the second stage, the achievable rate at the second stage can be determined by calculating the mutual information between $Z_{2}$ and $X_{2}$. By the definition of $f_{Q}$ in (2), $Z_{2}$ is given by

(6) $$\begin{array}{c} Z_{2}=\left\{\begin{array}{cc} 1, & X_{2}+W\geq 0\\ -1, & X_{2}+W<0 \end{array}\right.. \end{array}$$

Thus, if we define $Z_{3}$ as follows, the mutual information between $X_{2}$ and $Z_{2}$ is equal to the mutual information between $X_{2}$ and $Z_{3}$:

(7) $$\begin{array}{c} Z_{3}=f_{Q}\left(X_{2}+W,0\right). \end{array}$$

In other words, if we define $Z_{3}$ as the one-bit quantized value of $X_{2}+W$ obtained using a zero threshold for quantization, then the mutual information between $X_{2}$ and $Z_{2}$ is equal to the mutual information between $X_{2}$ and $Z_{3}$. The mutual information between the input signal and the one-bit quantized output with a zero threshold was derived in Theorem 2 by [16]. It can be expressed as $1-H_{b}\left(Q\left(\sqrt{E[|X_{2}{|}^{2}]}\right)\right)$, where $H_{b}\left(\cdot \right)$ represents the binary entropy function and Q(·) denotes the quantization function. □

From these lemmas, the achievable sum rate of the proposed scheme can be represented by a function of two variables $P_{1}$ and $P_{2}$ as follows:

(8) $$\begin{array}{cc} \; & R_{\mathrm{sum}}\left(P_{1},P_{2}\right)=2-H_{b}\left(Q\left(\sqrt{P_{2}}\right)\right)\\ \; & \;-H_{b}\left(\frac{Q(\sqrt{P_{1}}-\sqrt{P_{2}})}{2}+\frac{Q(\sqrt{P_{1}}+\sqrt{P_{2}})}{2}\right). \end{array}$$

In the following subsections, power allocation problems are considered when we have some transmit power constraints.

### 3.2. Individual Power Constraints

For simplicity, we call the two transmitters α and β, and denote their respective transmit signals as $X_{\alpha }$ and $X_{\beta }$. In this subsection, we consider the case when the two transmitters have individual power constraints $P_{\alpha }=E[|X_{\alpha }{|}^{2}]\leq P_{\alpha }^{U}$ and $P_{\beta }=E[|X_{\beta }{|}^{2}]\leq P_{\beta }^{U}$, where $P_{\alpha }^{U}$ and $P_{\beta }^{U}$ are independent deterministic constants. We must identify the case that achieves a higher achievable rate between the two cases $\left(X_{1}=X_{\alpha },X_{2}=X_{\beta }\right)$ and $\left(X_{1}=X_{\beta },X_{2}=X_{\alpha }\right)$; the former is the case where the signal of transmitter α is first detected, and the latter is the case where the signal of transmitter β is first detected (we assumed in the previous section that the signal denoted as $X_{1}$ is detected first). Considering that upper bounds $P_{\alpha }^{U}$ and $P_{\beta }^{U}$ are independent, we can assume that $P_{\alpha }^{U}\geq P_{\beta }^{U}$ without loss of generality.

Let $R_{\alpha }^{*}$ and $R_{\beta }^{*}$ be the maximum sum rates corresponding to the cases $\left(X_{1}=X_{\alpha },X_{2}=X_{\beta }\right)$ and $\left(X_{1}=X_{\beta },X_{2}=X_{\alpha }\right)$, respectively. For any given $P_{2}$, $R_{1}$ in (5) is an increasing function of $P_{1}$. Thus, we have

(9) $$\begin{array}{cc} R_{\alpha }^{*} & =\max_{0\leq P_{\beta }\leq P_{\beta }^{U}}R_{\mathrm{sum}}\left(P_{\alpha }^{U},P_{\beta }\right), \end{array}$$

(10) $$\begin{array}{cc} R_{\beta }^{*} & =\max_{0\leq P_{\alpha }\leq P_{\alpha }^{U}}R_{\mathrm{sum}}\left(P_{\beta }^{U},P_{\alpha }\right), \end{array}$$

and the maximum sum rate with individual power constraints is given as

(11) $$\begin{array}{cc} R_{\mathrm{indv}}^{*} & =\max(R_{\alpha }^{*},R_{\beta }^{*}). \end{array}$$

**Remark** **1.** The Gaussian Q-function can be closely approximated by a linear function as $Q\left(x\right)\approx -\frac{1}{\sqrt{2\pi }}x+\frac{1}{2}$ near x=0, such that if x and y are sufficiently small, we may use the approximation $\frac{Q(x+y)}{2}+\frac{Q(x-y)}{2}\approx Q\left(x\right)$, where the approximation error converges to zero as x and y approaches 0. Therefore, in a low SNR regime, we may approximate $R_{1}$ as $R_{1}\approx 1-H_{b}\left(Q\left(\sqrt{P_{1}}\right)\right)$, such that $R_{\alpha }^{*}$ and $R_{\beta }^{*}$ are achieved at $P_{\alpha }=P_{\alpha }^{U}$ and $P_{\beta }=P_{\beta }^{U}$ and have the same approximated values. Thus, $R_{indv}^{*}\approx R_{sum}\left(P_{\alpha }^{U},P_{\beta }^{U}\right)$.

**Remark** **2.** *In (5), if $P_{1}<P_{2}$, then $Q\left(\sqrt{P_{1}}-\sqrt{P_{2}}\right)\geq \frac{1}{2}$. Given that Q(x) is a fast-decreasing function, the rate $R_{sum}$ is rapidly saturated to an upper bound that is smaller than $2-H_{b}\left(\frac{1}{4}\right)\approx 1.1887$ when $P_{1}$ and $P_{2}$ increase. Hence, if $P_{1}$ and $P_{2}$ are moderately large, then detecting the signal with a higher transmit power is more advantageous, such that $R_{sum}$ can be increased up to 2 with increasing $P_{1}$ and $P_{2}$.*

Thus, Remark 1 implies that, in a low SNR regime, the detection order in the proposed scheme is not significant. In addition, Remark 2 suggests that in moderate and high SNR regimes, it is more advantageous to detect the transmit signal with higher transmit power at the first stage. That is, $\left(X_{1}=X_{\alpha },X_{2}=X_{\beta }\right)$ is generally preferred when $P_{\alpha }^{U}\geq P_{\beta }^{U}$.

Therefore, $R_{\mathrm{indv}}^{*}$ can be approximated as $R_{\mathrm{indv}}^{*}\approx R_{\alpha }^{*}=\max_{0\leq P_{\beta }\leq P_{\beta }^{U}}R_{\mathrm{sum}}\left(P_{\alpha }^{U},P_{\beta }\right)$. Finally, an optimal value of $P_{\beta }$ must be determined. However, $R_{\mathrm{sum}}\left(P_{\alpha }^{U},P_{\beta }\right)$ has multiple critical points with respect to $P_{\beta }$, making it difficult to mathematically determine the optimal value. Instead, we can numerically determine optimal $P_{\beta }$ through offline simulation in the AWGN channel. The corresponding results can be utilized as a lookup table to determine optimal values for wireless fading channels, as described in the following sections.

### 3.3. Total Power Constraint

In this subsection, we consider the case when the sum power is upper-bounded by a constant, such as $P_{\alpha }+P_{\beta }=P_{1}+P_{2}\leq P_{T}$. The maximization problem is defined as

(12) $$\begin{array}{cc} \; & R_{\mathrm{total}}^{*}=\max_{0\leq P_{1}+P_{2}\leq P_{T}}R_{\mathrm{sum}}\left(P_{1},P_{2}\right). \end{array}$$

**Figure 2:**
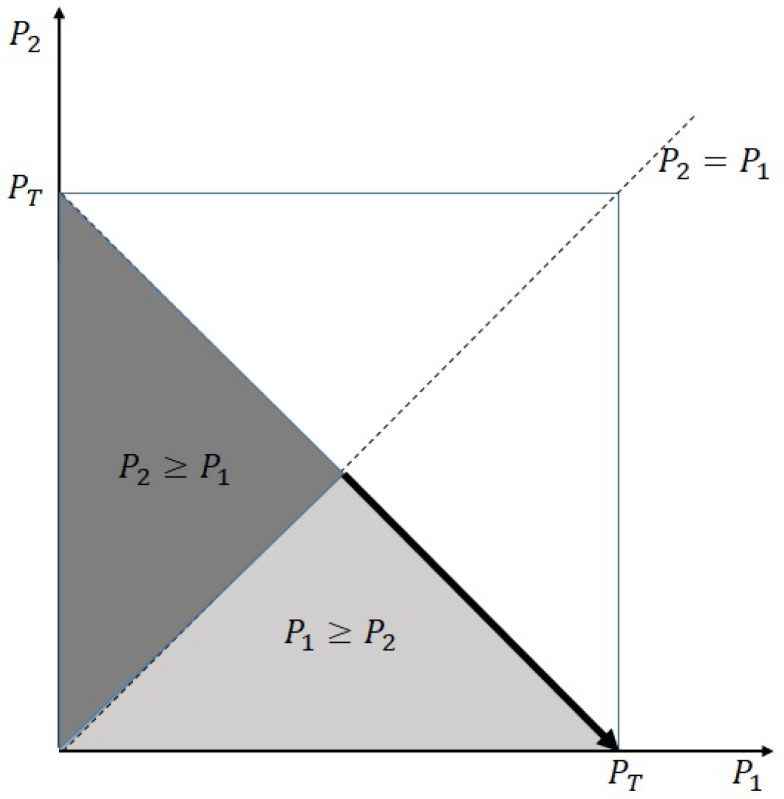
Domain for optimization.

The domain of interest for the maximization is given by the colored region (the union of light and dark gray regions) in Figure 2, whose boundary is described by $P_{1}\geq 0$, $P_{2}\geq 0$, and $P_{1}+P_{2}\leq P_{T}$. In the previous subsection, we explained that the first detection of the transmit signal with higher transmit power is more advantageous. Hence, we can claim that the optimal transmit powers $P_{1}^{*}$ and $P_{2}^{*}$ will exist in the region $P_{1}\geq P_{2}$ (light gray-colored region in Figure 2) with a high probability. If $P_{1}<P_{2}$ (within the dark gray-colored region), then the sum rate is less than the optimal rate (the rate is saturated to the upper bound of 1.1887, as discussed in Remark 2). For any given $P_{2}$, $R_{1}$ is an increasing function of $P_{1}$. Thus, the optimal value will exist in the rightmost boundary of the light-gray region and the optimization problem can be modified as

(13) $$\begin{array}{cc} R_{\mathrm{total}}^{*} & \approx \max_{P_{1}+P_{2}=P_{T},P_{1}\geq P_{2}}R_{\mathrm{sum}}\left(P_{1},P_{2}\right), \end{array}$$

where the region for maximization corresponds to the arrow depicted in Figure 2.

In practice, an optimal value of $P_{1}$ (for a given value of $P_{1}$, $P_{2}$ is also deterministic as $P_{2}=P_{T}-P_{1}$ at the optimal point) can be numerically obtained through offline simulation in the AWGN channel as a function of $P_{T}$. The corresponding result can be used as a lookup table to determine an optimal power allocation in realistic fading channels, as is demonstrated in the following section. This type of approach is similar to the usage of SNR versus block error rate lookup table (evaluated in the AWGN channel) to determine an appropriate modulation and coding set in long-term evolution standards.

## 4. Extension to Complex Fading Channels

In practice, a baseband digital communication channel is generally given by a complex fading channel. One of the most popular examples is the Rayleigh fading channel which is widely used to model wireless communication channels. In a narrow-band fading environment, two-transmitter and single-receiver AWGN MAC with fading can be expressed as

(14) $$\begin{array}{c} Y=H_{1}X_{1}+H_{2}X_{2}+W_{c}, \end{array}$$

where $H_{1}$ and $H_{2}$ are random variables that model fading channels between transmitters and the receiver. In the Rayleigh fading environment, $H_{1}$ and $H_{2}$ are modeled as complex circularly symmetric Gaussian random variables, and $W_{c}$ is complex white Gaussian noise that has zero mean and unit variance and is also circularly symmetric.

If the CSIs $H_{1}$ and $H_{2}$ are known at the transmitter, then transmitter can equalize the phase of the corresponding channel by transmitting $e^{-j\phi_{i}}X_{i}$, where $\phi_{i}$ denotes the phase of $H_{i}$, for i=1,2. Considering $X_{1}$ and $X_{2}$ are complex baseband signals, we can directly extend the proposed scheme to the rotated quadrature phase shift keying (QPSK) signaling for each $X_{i}$ (assuming individually imposing BPSK signals for real and imaginary parts) [17]. With QPSK signaling and phase equalization at the transmitter, the received signal can be rewritten as

(15) $$\begin{array}{c} Y=|H_{1}|X_{1}+\left|H_{2}\right|X_{2}+W_{c}. \end{array}$$

Here, we investigate the real and imaginary parts separately, which can be presented as follows:

(16) Re(Y)=|H1|Re(X1)+|H2|Re(X2)+Re(Wc)Im(Y)=|H1|Im(X1)+|H2|Im(X2)+Im(Wc),

where Re(·) and Im(·) represent the real and imaginary parts of a complex number, respectively. Thus, we have two equivalent real-valued channels from the complex baseband channel. Given that a QPSK signal has the same signal powers at real and imaginary parts, |Re(X1)|=|Im(X1)|=P1/2 and |Re(X2)|=|Im(X2)|=P2/2. In addition, the real and imaginary parts of $W_{c}$ have half-variance of $W_{c}$. Therefore, the real and imaginary channels in (16) can be represented as

(17) $$\begin{array}{cc} \; & Y^{R}=X_{1}^{R}+X_{2}^{R}+W^{R},\\ \; & Y^{I}=X_{1}^{I}+X_{2}^{I}+W^{I}, \end{array}$$

where $E\left[X_{1}^{R}\right]=E\left[X_{1}^{I}\right]=\frac{|H_{1}{|}^{2}P_{1}}{2}$, $E\left[X_{2}^{R}\right]=E\left[X_{2}^{I}\right]=\frac{|H_{2}{|}^{2}P_{2}}{2}$, and $E\left[W^{R}\right]=E\left[W^{I}\right]=\frac{1}{2}$. Then, we can individually apply Lemmas 1 and 2 for real ($Y^{R}$) and imaginary ($Y^{I}$) channels, respectively, to obtained the instantaneous sum rate of the proposed scheme. That is, the instantaneous sum rate of the proposed scheme with QPSK signaling for $X_{1}$ and $X_{2}$ is given by

(18) $$\begin{array}{cc} R_{\mathrm{fading}}\left(P_{1},P_{2},H_{1},H_{2}\right) & =4-2H_{b}\left(Q(|H_{2}|\sqrt{P_{2}})\right)\\ \; & \;-2H_{b}\left(\frac{Q(|H_{1}|\sqrt{P_{1}}-|H_{2}|\sqrt{P_{2}})+Q(|H_{1}|\sqrt{P_{1}}+|H_{2}\left|\sqrt{P_{2}}\right)}{2}\right). \end{array}$$

The transmit power multiplied by channel gain $|H_{i}{|}^{2}$ can be regarded as an effective SNR. If we have a total power constraint, then the optimization problem is derived from (12) and (18) as:

(19) $$\begin{array}{cc} R_{\mathrm{total}}^{*} & =\max_{0\leq P_{1}+P_{2}\leq P_{T}}2R_{\mathrm{sum}}\left(\right|H_{1}{|}^{2}P_{1},|H_{2}{|}^{2}P_{2}). \end{array}$$

## 5. Numerical Results

### 5.1. AWGN MAC without Channel Fading

**Figure 3:**
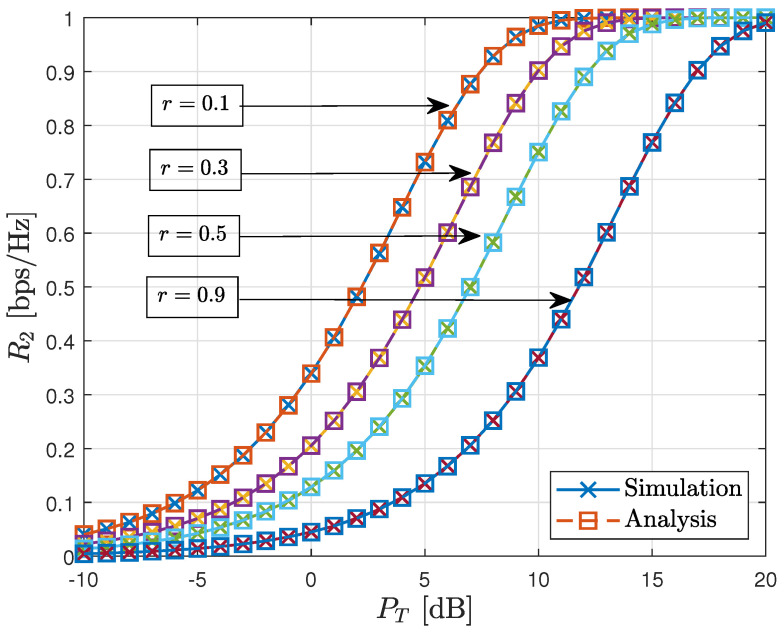
Verification for Lemma 2.

In this subsection, we present simulation results for the AWGN MAC without fading, as described in (1). Figure 3 serves as a verification of Lemma 2, which was discussed in Section 3. The parameter *r* in the figure represents the transmit power ratio, defined as $r=P_{1}/P_{T}$, where $P_{2}=\left(1-r\right)P_{T}$. The dashed lines with square markers (labeled as “Analysis” in the figure) depict the graphs of the analysis results, specifically $R_{2}=1-H_{b}\left(Q\left(\sqrt{P_{2}}\right)\right)$, as derived in Lemma 2. On the other hand, the solid lines with cross markers (labeled as “Simulation” in the figure) represent the average mutual information between $Z_{2}=f_{Q}\left(X_{1}+X_{2}+W,X_{1}\right)$ and $X_{2}$, which quantifies the achievable rate at the second stage. The simulation results align with the analysis results as presented in Lemma 2, irrespective of the power ratio between the transmitters.

**Figure 4:**
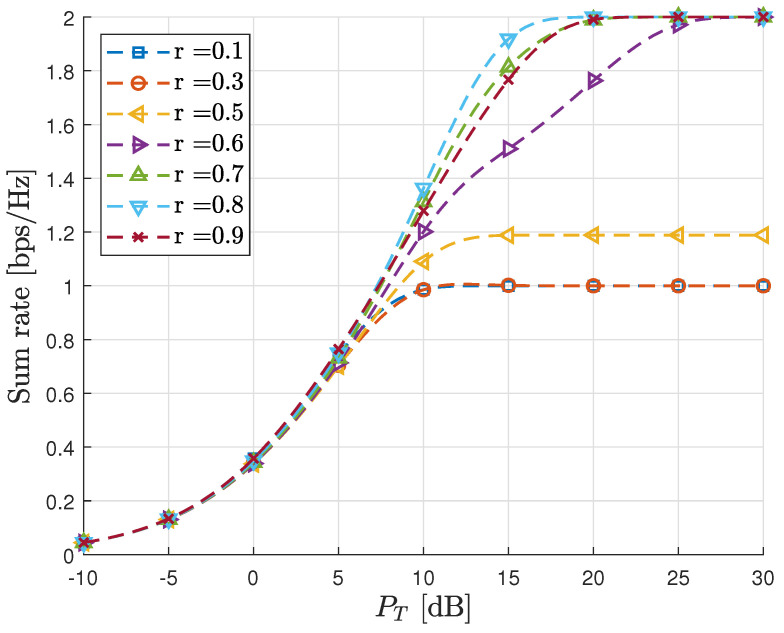
Sum rate vs. $P_{T}$ in AWGN MAC.

In Figure 4, we present the achievable sum rates of the proposed scheme in the AWGN MAC for various power ratios (*r*). As anticipated in Remark 2, it is observed that at high SNR, the maximum achievable sum rate is 2 bps/Hz when the power ratio *r* is appropriately selected. In the figure, it can be generally observed that for a given total transmit power ($P_{T}$), the maximum sum rate is attained when r≥0.5, as anticipated in Section 3.3. It is important to note that the optimal value of *r* varies depending on the total transmit power.

**Figure 5:**
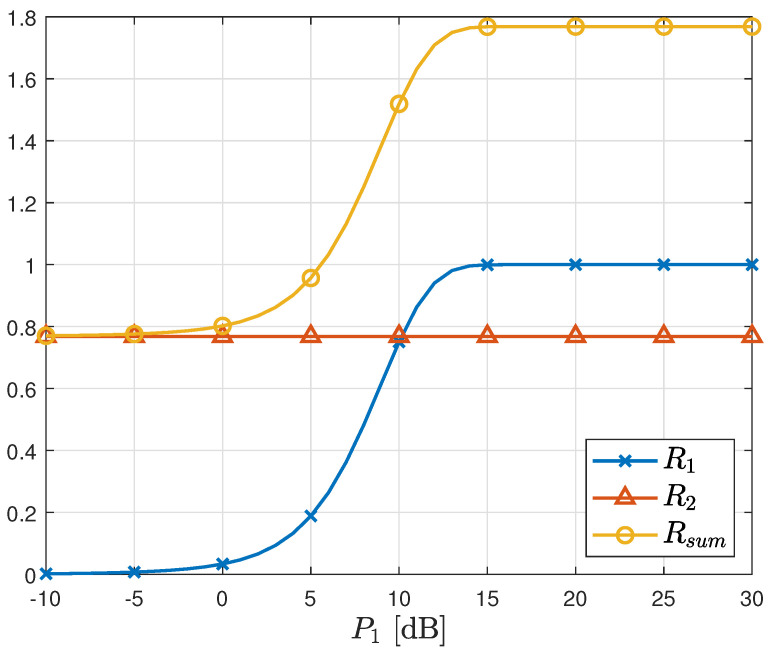
Sum rate vs. $P_{1}$ in AWGN MAC. $P_{2}=5$ [dB].

**Figure 6:**
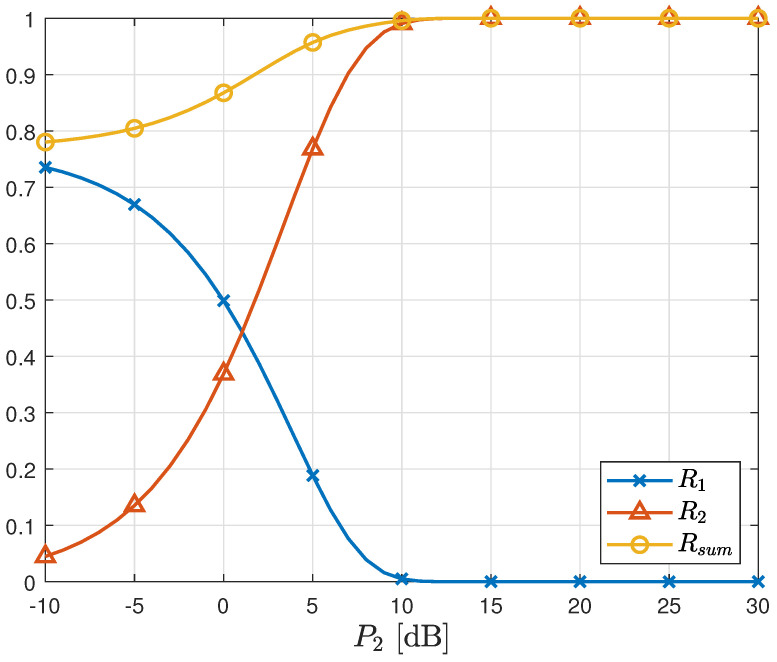
Sum rate vs. $P_{2}$ in AWGN MAC. $P_{1}=5$ [dB].

Figure 5 illustrates the sum rate of the proposed scheme as a function of $P_{1}$, with $P_{2}$ fixed at 5 dB. The rate $R_{2}$ remains independent of $P_{1}$ due to threshold adaptation, which eliminates interference from $X_{1}$. Consequently, the sum rate increases with increasing $P_{1}$. In contrast, Figure 6 presents the sum rate of the proposed scheme as a function of $P_{2}$, with $P_{1}$ fixed at 5 dB. Unlike the case in Figure 5, the rate $R_{1}$ is influenced by $P_{2}$, as interference cancellation is not considered in the first stage detection. As a result, the sum rate is significantly lower than that achieved in Figure 5, even though the same total transmit power is used. This observation indirectly highlights the greater importance of the first stage detection.

### 5.2. AWGN MAC with Channel Fading

In this subsection, we present simulation results obtained in the AWGN MAC with complex channel fading, as defined in Section 4 of this study. As depicted in Figure 4, the sum rate of the proposed method is found to be sensitive to the power ratio between the received signals, calculated as $P_{1}\;\;:\;\;P_{2}$ in the AWGN MAC. Consequently, in the case of the AWGN MAC, an optimal power ratio can be determined through offline simulations conducted over a suitable range of $P_{T}$ with adequate resolution. However, when considering fading channels, the received signal power ratio, calculated as $|H_{1}{|}^{2}P_{1}\;\;:\;\;{\left|H_{2}\right|}^{2}P_{2}$, is also influenced by the channel gains. Since the range of $H_{i}$ is the set of positive real numbers, it is practically infeasible to perform offline simulations for all possible combinations of the ratio $|H_{1}{|}^{2}P_{1}:{\left|H_{2}\right|}^{2}P_{2}$. Therefore, an optimal power allocation strategy that is applicable in practical scenarios is required to address the challenges associated with fading channels.

For simplicity, the variances of $H_{1}$ and $H_{2}$ are normalized to 1 in this subsection. To obtain the optimal power allocation in fading channels, we use a lookup table-based approach, as described in the previous sections. The lookup is constructed through an offline simulation in the AWGN channel described in Section 2 and Section 3. For simple description, the power ratio *r* is defined such that $P_{1}=rP_{T}$ and $P_{2}=\left(1-r\right)P_{T}$. We want to determine an optimal power ratio for each realization of channel values using the AWGN lookup table. The lookup table saves the sum rate $R_{\mathrm{sum}}$ as a function of two variables *r* and $P_{T}$. In this simulation, the lookup table is established under the assumptions 0≤r≤1, $-30\leq 10\log_{10}P_{T}\leq 100$, and their quantization precision is 0.1.

Then, we use the lookup table to determine the optimal value of $P_{1}$ in (19) for given $H_{1},H_{2},$ and $P_{T}$ as follows:

1. Using the same values of *r* with the lookup table, we use $P_{1}=rP_{T}$ and $P_{2}=\left(1-r\right)P_{T}$ as candidates for optimal power allocation. However, this ratio cannot be directly used as an input for the lookup table. This is because, the effective powers (transmit powers multiplied by the channel gains) are used as input values for $R_{\mathrm{sum}}$ in fading channels as described in (19), which are different from those in the AWGN channel. Thus, we need to calculate the effective power ratio: $t_{1}=\frac{|H_{1}{|}^{2}P_{1}}{|H_{1}{|}^{2}P_{1}+{\left|H_{2}\right|}^{2}P_{2}}$ and $t_{2}=\frac{|H_{2}{|}^{2}P_{2}}{|H_{1}{|}^{2}P_{1}+{\left|H_{2}\right|}^{2}P_{2}}$ for each *r*, and then use these ratios as an input for the lookup table.
2. Similarly, the input SNR for the lookup table must also be modified to an effective value. For given $H_{1},H_{2},$ and $P_{T}$, the maximum effective SNR is given by $P_{eff}=10\log_{10}\left(\right|H_{1}{|}^{2}P_{1}+|H_{2}{|}^{2}P_{2})$ in decibel scale.
3. For each *r*, the effective ratios $t_{1}$, $t_{2}$, and the effective SNR (in dB) are now obtained. By using two pairs $\left(t_{1},P_{eff}\right)$ and $\left(t_{2},P_{eff}\right)$ as input pairs for the lookup table, we can obtain two expected sum rates for each *r*. The value of *r* that corresponds to the maximum value of $R_{\mathrm{sum}}$ provides the optimal power allocation.

**Figure 7:**
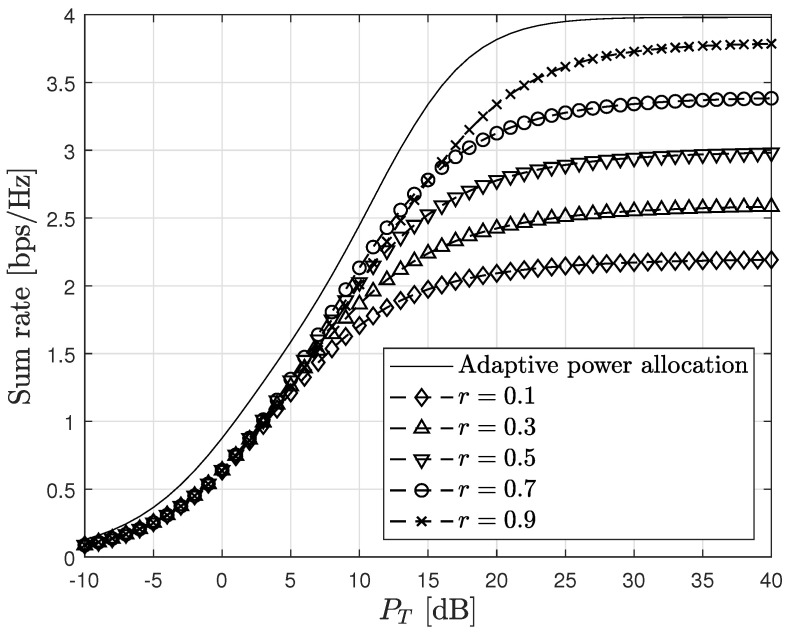
Adaptive power allocation for fading channels.

Figure 7 demonstrates the advantage of determining optimal power allocation for each channel realization. This type of adaptive power allocation achieves a considerable gain compared with the cases using fixed power ratios which is independent of channel realizations. In Figure 7, each dashed line represents the sum rates ($R_{\mathrm{sum}}$) obtained using a fixed power ratio (*r*) selected from the set 0.1,0.3,0.5,0.7,0.9. As observed in the figure, the sum rate increases with *r* at very high SNR, but this trend diminishes at moderate SNR levels. Thus, it is evident that an appropriate power allocation strategy can further enhance the achievable rate, as previously discussed in Figure 4. The solid line in the figure represents the sum rate achieved using the proposed adaptive power allocation algorithm. By selecting an optimal power distribution based on the given channel realizations, the proposed algorithm globally achieves the maximum sum rate across various SNR levels.

**Figure 8:**
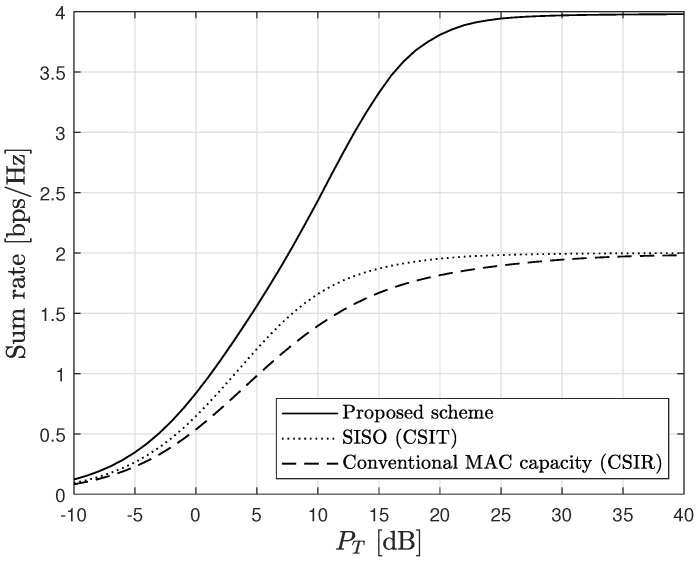
Comparison with conventional results.

Figure 8 compares the performance of the proposed scheme with the conventional scheme [32], where the conventional scheme represents the achieved capacity assuming perfect CSIR in normal two-transmitter MAC (4) with one-bit ADCs at the receiver. The proposed scheme can achieve twofold higher spectral efficiency than the conventional scheme and the SISO channel capacity [17] at high SNR because it can successfully remove non-orthogonal interference in MAC. In contrast, the spectral efficiency of the conventional scheme is upper-bounded by the same limit as the SISO channel capacity, due to the non-orthogonal interference, which cannot be eliminated in the DSP domain when one-bit ADCs are used at the receiver.

The conventional scheme [32] in Figure 8 (dashed line, which depicts (4)) is derived considering perfect CSI only at the receiver. Therefore, the corresponding spectral efficiency is smaller than the SISO capacity derived assuming perfect CSIT (dotted line, which depicts (3)). Nevertheless, we use this conventional scheme [32] for comparison purposes because the capacity achieving signaling with perfect CSIT is unknown [31]. Although the gap in the amount of available CSIT imposes a disadvantage to the conventional scheme, the important thing is that the capacity available in the normal MAC structure is saturated to the same upper bound with the SISO capacity when one-bit ADCs are used at the receiver, regardless of the amount of CSIT [31]. Nonlinear ISI between different transmitters cannot sufficiently be mitigated with the conventional MAC structure when the receiver uses one-bit ADCs. The saturation phenomenon observed in the conventional normal MAC structure is inevitable even with perfect CSIT [31], because the presence of nonlinear ISI induced by one-bit quantization in the receiving RF chain is independent of CSIT. The proposed scheme overcomes this limitation by employing analog domain threshold adaptation, which effectively mitigates the nonlinear ISI mentioned above. As a result, the proposed scheme achieves a twofold higher sum rate compared to conventional methods.

## 6. Conclusions

In this study, we propose a novel analog-domain interference cancellation scheme for a two-transmitter MAC with one-bit ADCs. Unlike the conventional MAC structure, our proposed scheme enables the implementation of a non-orthogonal multiple access structure that is compatible with one-bit ADCs through the use of an appropriate threshold adaptation-based interference cancellation method. With our proposed scheme, the corresponding two-transmitter MAC structure achieves a twofold higher spectral efficiency, even when the total transmit power is fixed. This increase is significant because it was previously unattainable using digital-domain signal processing due to the detrimental nonlinear distortion introduced by one-bit ADCs, which severely affects interference cancellation. However, our proposed scheme overcomes this limitation by applying a novel threshold adaptation technique in the analog domain.

In our study, we initially demonstrated that the capacity of a one-bit-quantized channel with fixed interference can be made equivalent to the capacity of a one-bit-quantized channel without interference by appropriately setting the threshold value equal to the interference level. This result allowed us to perform successive detection of the two transmitter’s signals in a sequential manner. While the first detected signal may experience some interference from the other signal, the second detection can be performed without interference, assuming that the first detection is accurate. This sequential detection approach led us to conclude that the accuracy of the first stage detection is more crucial than the second detection. We took this insight into account when deriving an optimal power allocation strategy while satisfying the total power constraint. The main advantage of our proposed scheme stems from its ability to effectively mitigate non-orthogonal interference between the two transmit signals. By exploiting the successive interference cancellation capability, our scheme enables improved signal detection and communication performance. Additionally, our study revealed that the achievable rate of the proposed scheme is highly dependent on the power ratio between the two transmitters, especially in wireless fading environments. To tackle this challenge, we derived an appropriate power allocation strategy aimed at maximizing the achievable sum rate. The proposed power allocation algorithm is based on a lookup table approach, which is constructed through offline simulations in AWGN channels. The algorithm determines an optimal power allocation for given realizations of channel gains, leading to a significant increase in the sum rate of the proposed system. This approach allows us to adapt the power allocation dynamically based on the specific channel conditions, thereby maximizing the overall system performance.

Through a combination of simulations and analysis, our study demonstrated that the proposed scheme, along with its accompanying power allocation strategy, leads to a substantial enhancement in the total spectral efficiency compared to conventional schemes. This improvement offers a promising approach to enhance the performance of a two-transmitter Gaussian MAC system utilizing one-bit ADCs. Furthermore, the results obtained in this study can serve as a foundation for extending the proposed scheme to scenarios involving multiple transmitters beyond just two. In the case of multiple transmitters, it is expected that the achievable sum rate will increase linearly with the number of transmitters. However, this would require the utilization of additional RF chains to facilitate the proposed threshold adaptation process. By employing more RF chains, the system can effectively manage the interference and achieve higher overall spectral efficiency. It is important to note that the scalability of the proposed scheme to multiple transmitters introduces additional complexity in terms of hardware requirements and implementation considerations. However, the potential benefits in terms of increased sum rate make it a worthwhile avenue for further exploration and research.

## Data Availability

Not applicable.

## Appendix A. Proof of Lemma 1

From the relation between the capacity and mutual information, $R_{1}$ is given by

(A1) $$\begin{array}{c} R_{1}=I\left(X_{1};Z_{1}\right)=H\left(Z_{1}\right)-H\left(Z_{1}|X_{1}\right), \end{array}$$

where $Z_{1}=f_{Q}\left(X_{1}+X_{2}+W,0\right)$ and H(·) denotes the entropy of a random variable. Given that $Z_{1}$ is binary and $X_{1}$, $X_{2}$ and *W* are symmetric, we have $H(Z_{1})=1$. Furthermore,

(A2) $$\begin{array}{cc} H(Z_{1}|X_{1}) & =E[H_{b}\left(p_{1}\left(X_{1}\right)\right)], \end{array}$$

where $p_{1}\left(x\right)=\mathrm{Pr}\left(Z_{1}=1|X_{1}=x\right)$ and calculated as:

(A3) $$\begin{array}{cc} p_{1}\left(x_{1}\right) & =\mathrm{Pr}\left(Z_{1}=1|X_{1}=x_{1}\right)=\mathrm{Pr}\left(Z_{1}>0|X_{1}=x_{1}\right)\\ \; & =\mathrm{Pr}(W+x_{1}+X_{2}>0)\\ \; & =\sum_{x_{2}\in X_{2}}\mathrm{Pr}\left(X_{2}=x_{2}\right)\mathrm{Pr}\left(-W<x_{1}+x_{2}\right)\\ \; & =\sum_{x_{2}\in X_{2}}\mathrm{Pr}\left(X_{2}=x_{2}\right)\left(1-Q\left(x_{1}+x_{2}\right)\right)\\ \; & =1-E[Q\left(x_{1}+X_{2}\right)]. \end{array}$$

By substituting (A3) into (A2), we obtain

(A4) $$\begin{array}{cc} H(Z_{1}|X_{1}) & \overset{\left(a\right)}{=}E_{X_{1}}\left[H_{b}\left(E_{X_{2}}\left[Q\left(X_{1}+X_{2}\right)\right]\right)\right]\\ \; & =E_{X_{1}}\left[H_{b}\left(\sum_{x_{2}\in X_{2}}\mathrm{Pr}\left(X_{2}=x_{2}\right)Q\left(X_{1}+x_{2}\right)\right)\right]\\ \; & =\sum_{x_{1}\in X_{1}}\left\{\mathrm{Pr}\left(X_{1}=x_{1}\right)H_{b}\left(\sum_{x_{2}\in X_{2}}\mathrm{Pr}\left(X_{2}=x_{2}\right)Q\left(x_{1}+x_{2}\right)\right)\right\}, \end{array}$$

where (a) follows because $H_{b}\left(1-x\right)=H_{b}\left(x\right)$ and $X_{1}$ and $X_{2}$ represent the sample spaces of $X_{1}$ and $X_{2}$, respectively.

Given that $X_{1}$ and $X_{2}$ are obtained by BPSK signaling with average signal powers $P_{1}$ and $P_{2}$, respectively, we have $X_{1}=\left\{-\sqrt{P_{1}},\sqrt{P_{1}}\right\}$, $X_{2}=\left\{-\sqrt{P_{2}},\sqrt{P_{2}}\right\}$, and $\mathrm{Pr}\left(X_{i}=-\sqrt{P_{i}}\right)=\mathrm{Pr}\left(X_{i}=\sqrt{P_{i}}\right)=1/2$, i=1,2. Therefore,

(A5) $$\begin{array}{cc} H(Z_{1}|X_{1}) & =\sum_{x_{1}\in X_{1}}\left\{\frac{1}{2}H_{b}\left(\frac{Q(x_{1}-\sqrt{P_{2}})}{2}+\frac{Q(x_{1}+\sqrt{P_{2}})}{2}\right)\right\}\\ \; & =\frac{1}{2}H_{b}\left(\frac{Q(-\sqrt{P_{1}}-\sqrt{P_{2}})}{2}+\frac{Q(-\sqrt{P_{1}}+\sqrt{P_{2}})}{2}\right)\\ \; & \;\;+\frac{1}{2}H_{b}\left(\frac{Q(\sqrt{P_{1}}-\sqrt{P_{2}})}{2}+\frac{Q(\sqrt{P_{1}}+\sqrt{P_{2}})}{2}\right). \end{array}$$

Given that Q(x)=1−Q(−x) and $H_{b}\left(1-x\right)=H_{b}\left(x\right)$, two terms on the right-hand side of (A5) are equal, such that

(A6) $$\begin{array}{cc} H(Z_{1}|X_{1})\; & =H_{b}\left(\frac{Q(\sqrt{P_{1}}-\sqrt{P_{2}})}{2}+\frac{Q(\sqrt{P_{1}}+\sqrt{P_{2}})}{2}\right). \end{array}$$

Thus, the proof is completed by substituting (A6) into (A1).
